# Evaluating the impact of a hand-crafted 3D-Printed head Model and virtual reality in skull base surgery training

**DOI:** 10.1016/j.bas.2024.104163

**Published:** 2024-12-12

**Authors:** Amine Mellal, Pablo González-López, Lorenzo Giammattei, Mercy George, Daniele Starnoni, Giulia Cossu, Jan Frederick Cornelius, Moncef Berhouma, Mahmoud Messerer, Roy Thomas Daniel

**Affiliations:** a Department of Neurosurgery, University Hospital of Lausanne and University of Lausanne, 1011, Lausanne, Switzerland; b Department of Neurosurgery, General University Hospital Alicante and University of Alicante, Spain; c Department of ENT and Head and Neck Surgery, University Hospital of Lausanne, 1011, Lausanne, Switzerland; d Department of Neurosurgery, University Hospital of Düsseldorf, Heinrich Heine University, Düsseldorf, Germany; e Department of Neurosurgery, University Hospital of Dijon Bourgogne, 21000, Dijon, France; f Lundin Family Brain Tumour Research Centre, University Hospital of Lausanne, Switzerland

**Keywords:** Skull base surgery, 3D-printed model, Virtual reality, Neurosurgical education

## Abstract

**Introduction:**

While cadaveric dissections remain the cornerstone of education in skull base surgery, they are associated with high costs, difficulty acquiring specimens, and a lack of pathology in anatomical samples. This study evaluated the impact of a hand-crafted three-dimensional (3D)-printed head model and virtual reality (VR) in enhancing skull base surgery training.

**Research question:**

How effective are 3D-printed models and VR in enhancing training in skull base surgery?

**Materials and methods:**

A two-day skull base training course was conducted with 12 neurosurgical trainees and 11 faculty members. The course used a 3D-printed head model, VR simulations, and cadaveric dissections. The 3D model included four tumors and was manually assembled to replicate tumor-modified neuroanatomy. Trainees performed surgical approaches, with pre- and post-course self-assessments to evaluate their knowledge and skills. Faculty provided feedback on the model's educational value and accuracy. All items were rated on a 5-point scale.

**Results:**

Trainees showed significant improvement in understanding spatial relationships and surgical steps, with scores increasing from 3.40 ± 0.70 to 4.50 ± 0.53 for both items. Faculty rated the educational value of the model with a score of 4.33 ± 0.82, and a score of 5.00 ± 0.00 for recommending the 3D-printed model to other residents. However, realism in soft tissue simulations received lower ratings.

**Discussion and conclusion:**

Virtual reality and 3D-printed models enhance anatomical understanding and surgical training in skull base surgery. These tools offer a cost-effective, realistic, and accessible alternative to cadaveric training, though further refinement in soft tissue realism is needed.

## Highlights

• Hand-crafted 3D-printed head model replicating tumor-modified neuroanatomy.
• 3D model and VR used in a hands-on skull base course.
• Assessment of 3D and VR models' impact on anatomy and surgical skill learning.
• Significant improvement in trainees' understanding of anatomy and surgical steps.

## Abbreviations

3D: Three-dimensional
NET-Lab: Neurosurgery education and training laboratory
DICOM: Digital imaging and communication in medicine
CT: Computed tomography
MRI: Magnetic resonance imaging
IONM: Intraoperative neurophysiological monitoring
VR: Virtual reality
HMD: Head-mounted device
CTA: Computed tomography angiography
STL: Standard triangulated language
PLA: Polylactic acid
ABS: Acrylonitrile butadiene styrene
PETG: Polyethylene terephthalate glycol
SLA: Stereolithography
DLP: Digital light processing
CUSA: Cavitronic ultrasonic surgical aspirator
MCQ: Multiple choice question
AI: Artificial intelligence
SD: Standard deviation

## 1 Background

Advancements in neurosurgical education are essential to improving surgical outcomes, particularly in complex areas such as skull base surgery (Scholz et al., 2010). Traditional training methods, including cadaveric dissection, have long been the gold standard for developing surgical skills. However, these methods have limitations, such as high costs, the lack of pathology in the anatomical samples, and the institutional challenges associated with acquiring cadaveric specimens (Rajasekhar and Dinesh, 2021). To address these issues, innovative educational tools have been developed, including the use of 3D-printed models (Bartikian et al., 2019; Baskaran et al., 2016). These models offer an opportunity for trainees to practice and refine their skills in a controlled environment, replicating the anatomical and pathological characteristics of real clinical cases. The ability to simulate surgeries on lifelike models allows for a more realistic and practical learning experience, bridging the gap between theoretical knowledge and clinical practice (Ganguli et al., 2018)

**Fig. 1:**
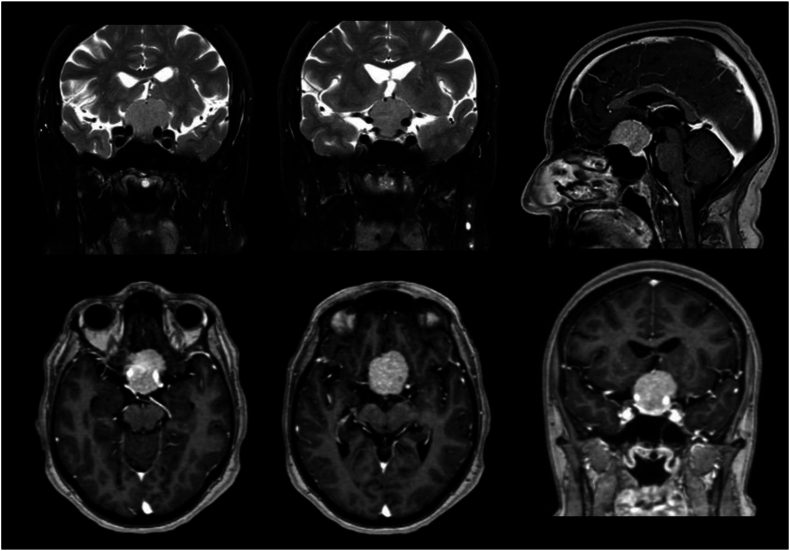
Pre-operative T1-Gd- and T2-weighted imaging showing a tuberculum sellae meningioma. These DICOM data were used to segment and create the 3D-models used for VR and 3D printing.

The first Lausanne NET-Lab (Neurosurgery Education and Training Laboratory) Skull Base Course, held at Lausanne University Hospital, Switzerland, in May 2024, was designed to utilize these advancements in neurosurgical training. The course provided hands-on experience using a combination of a hand-crafted head model using 3D printing, VR, and cadaveric dissections. The hand-crafted 3D-printed model accurately reflected the anatomy and pathology of 4 skull base tumors in the same head model, including *tuberculum sellae meningioma*, *falcotentorial meningioma*, *petroclival chordoma*, and *vestibular schwannoma*, and the adjacent neurovascular structures were accordingly modified. The model was built using digital imaging and communication in medicine (DICOM) data from computed tomography (CT) scans and magnetic resonance imaging (MRI) of actual patients (Fig. 1). Additionally, an electrical system with integrated light and sound was incorporated into the 3D-printed model to accurately replicate the use of intraoperative neurophysiological monitoring (IONM). This mix of training methods aimed to enhance the trainees' understanding of complex skull base surgeries through step-by-step simulations, guided by expert faculty. Additionally, the 3D model was incorporated into a VR environment on a head-mounted device (HMD) to further analyze the anatomy of the tumors and the surgical steps, adding another layer of depth to the training.

**Fig. 2:**
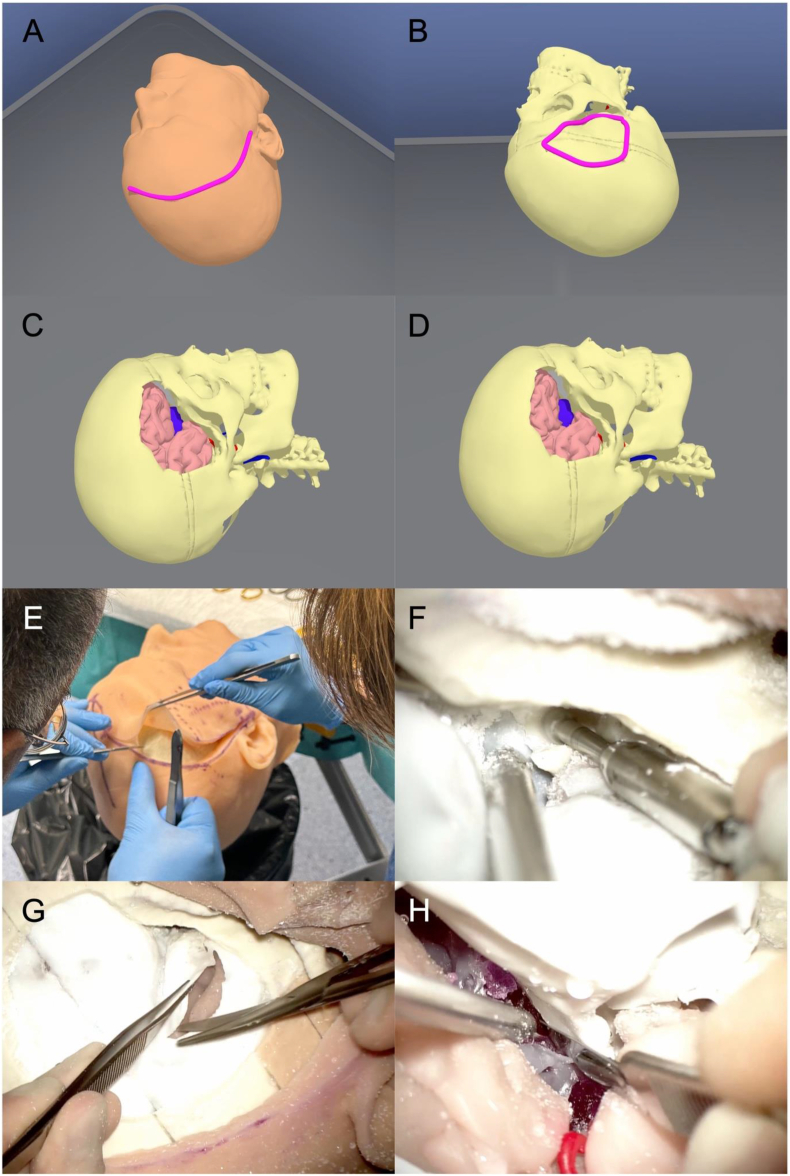
VR and 3D-model dissection of a tuberculum sellae meningioma. (A) Skin incision in the VR environment. (B) VR pterional craniotomy planning. (C) VR pterional craniotomy completed. (D) VR tumor visualization after sphenoid wing drilling, anterior clinoidectomy, and right frontal lobe retraction. (E) Skin incision on the 3D-printed model. (F) Extradural anterior clinoidectomy on the 3D-printed model. (G) Dural incision on the 3D-printed model. (H) Tumor debulking on the 3D-printed model.

By integrating these cutting-edge educational tools, the course aimed to evaluate their effectiveness through feedback obtained from both trainees and faculty. This paper presents a detailed description of the course and analyzes the results of the questionnaires filled out by participants, providing insights into the efficacy of our hand-crafted 3D-printed model and VR in neurosurgical education.

## 2 Methods

### 2.1 Study design

This prospective observational study evaluated the effectiveness of a multimodal skull base surgery hands-on training course, integrating a hand-crafted 3D-printed pathological head model, VR simulations, and traditional cadaveric dissections. The primary objective was to assess the educational value, realism, and feasibility of these combined educational tools in improving surgical training, with an emphasis on our 3D-printed model.

### 2.2 Participants

The course was attended by a total of 23 participants, including 12 neurosurgical trainees and 11 faculty members from various European institutions. The trainees were selected based on their interest and involvement in skull base surgery. The course faculty comprised both local and international experts in skull base surgery.

### 2.3 Educational tools and formats

1. Lectures

The course spanned two days, with each tumor and its surgical approach studied during each half-day session. Before the hands-on simulations, trainees attended lectures delivered by the expert faculty, who provided detailed didactic explanations of the surgical approaches to each of the tumors and outlined the key anatomical landmarks and technical surgical steps specific to these complex pathologies (Table 1). The tumors and their corresponding approaches in the course were.

- *Tuberculum sellae meningioma* (Fig. 1, Fig. 2): anterolateral skull base approach
- *Petroclival chordoma*: anterior petrosal approach
- *Falcotentorial meningioma*: occipito-transtentorial and supracerebellar infratentorial approaches
- *Vestibular schwannoma*: retrosigmoid and translabyrinthine approaches

**Table 1:** Surgical steps performed by faculty during the cadaveric head masterclass, followed by trainees on the 3D-printed model.

**Tuberculum sellae meningioma: anterolateral skull base approach** 1) Patient positioning 2) Skin incision 3) Pterional craniotomy. Including burr-hole(s) placement & dural identification 4) Sphenoid wing drilling 5) Extradural dissection 6) Identification of the dorsal surface of the anterior clinoid process, optic strut & optic canal 7) Extradural anterior clinoidectomy 8) Dural opening 9) Identification of the interoptic space, carotid artery and tumor 10) Tumor debulking with anterior circulation vessels preservation 11) Identification of the contralateral carotid artery and infundibulum 12) Optic canals exploration	**Petroclival chordoma: anterior petrosal approach** 1) Patient positioning 2) Skin incision 3) Subtemporal craniotomy. Including burr-hole(s) placement & dural identification 4) Basitemporal drilling 5) Extradural dissection 6) Identification of the middle meningeal artery and greater petrosal superficial nerve (including stimulation) 7) Interdural dissection to expose the petrous apex 8) Kawase space identification with boundaries definition 9) Drilling of the petrous apex defining the limits namely the posterior fossa dura, internal auditory meatus and the petrous ridge 10) Identification of the Glasscock triangle and petrous carotid artery 11) Ligation of the superior petrosal sinus, dural opening and Meckel's cave opening 12) Tumor excision 13) Identification of Vth, VIth, VII/VIIIth nerves and basilar artery
**Falcotentorial meningioma: occipital transtentorial approach** 1) Patient positioning 2) Skin incision 3) Anatomical landmarks identification (sutures) 4) Occipital craniotomy. Including burr-hole(s) placement & dural identification 5) Exposure of the posterior sagittal sinus, medial transverse sinus and torcula 6) Dural opening 7) Development of the interhemispheric corridor 8) Ipsilateral tentorial incision 9) Falcine incision 10) Transfalcine contralateral tentorial incision 11) Disconnection of the tumor from the sinus rectus 12) Tumor excision 13) Visualization of the vein of Galen, internal cerebral veins and splenium	**Falcotentorial meningioma: supracerebellar infratentorial approach** 1) Patient positioning 2) Skin incision (*sitting vs. lateral*). Surface marking for transverse sinus 3) Anatomical landmarks identification (inion) 4) Midline suboccipital craniotomy. Including burr-hole(s) placement & dural identification 5) Exposure of the transverse sinuses and torcula 6) Dural opening and ligation of the occipital sinus 7) Development of the supracerebellar corridor including the precentral cerebellar vein 8) Tumor identification 9) Tumor detachment from the falcotentorial attachment 10) Tumor excision 11) Visualization of the vein of Galen, internal cerebral veins, basal veins of Rosenthal, midbrain and splenium
**Vestibular schwannoma: retrosigmoid approach** 1) Patient positioning 2) Skin incision 3) Anatomical landmarks identification (asterion) 4) Retrosigmoid craniotomy. Including burr-hole(s) placement & dural identification 5) Exposing the transverse sinus and posterior part of the sigmoid sinus by drilling 6) Dural opening 7) Identification of the lateral cerebellomedullary cistern 8) Lower cranial nerves identification 9) Exposure of the dorsal surface of the tumor and surface nerve mapping 10) Tumor debulking and dissection of the capsule aided by facial nerve stimulation 11) Internal acoustic meatus opening 12) Tumor excision 13) Identification of IVth, Vth, VIth, VIIth nerves, and superior, anteroinferior and posteroinferior cerebellar arteries	**Vestibular schwannoma: translabyrinthine approach** 1) Patient positioning 2) Skin incision 3) Anatomical landmarks identification (spine of Henle, mastoid tip, posterior root of zygoma, triangle of attack) 4) Mastoid surface drilling 5) Identification of the mastoid antrum and lateral semicircular canal and incus 6) Mastoid segment of the facial nerve identification 7) Labyrinthectomy 8) Trautmann's triangle and internal acoustic meatus identification 9) Exposure of tegmen dura, presigmoid dura and superior petrosal sinus 10) Durotomy and visualization of the tumor within the meatus and cerebellopontine angle

Moreover, intraoperative videos of actual surgeries (for these tumors) and of simulations on the hand-crafted 3D-printed model were also shown and described to the trainees.

2. Segmentation and 3D-Modeling

**Fig. 3:**
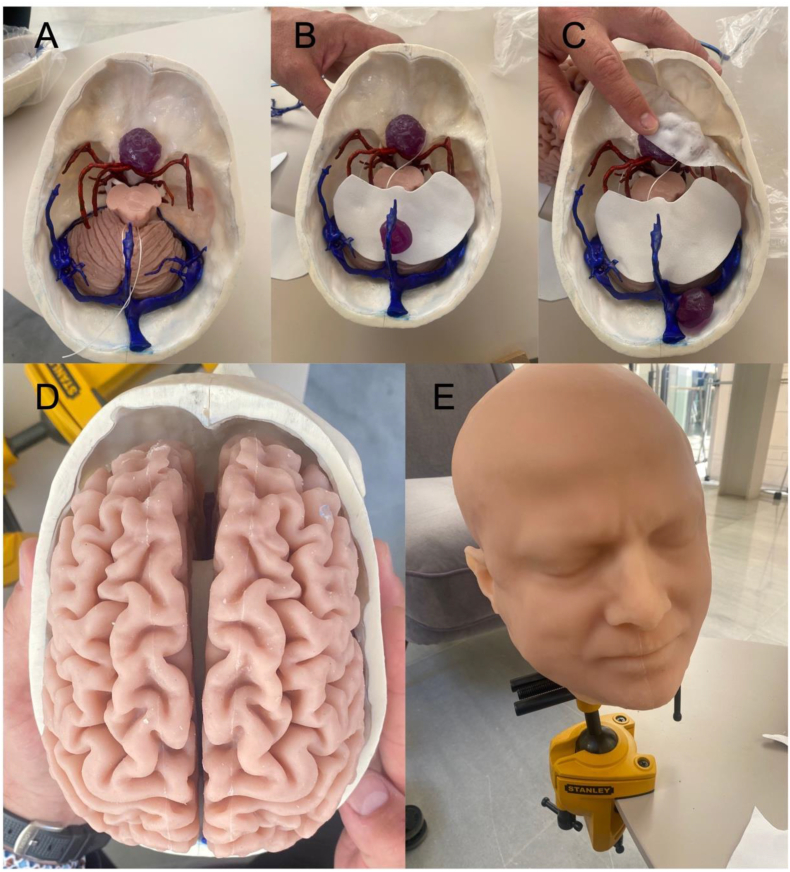
Sequential assembly of a prototype of our hand-crafted 3D-printed model. (A) Bone, brainstem and cerebellum parenchyma, arteries, sinuses and veins, tuberculum sellae meningioma. (B) Tentorium. (C) Basal dura. (D) Brain parenchyma. (E) Final external appearance with skin.

**Fig. 4:**
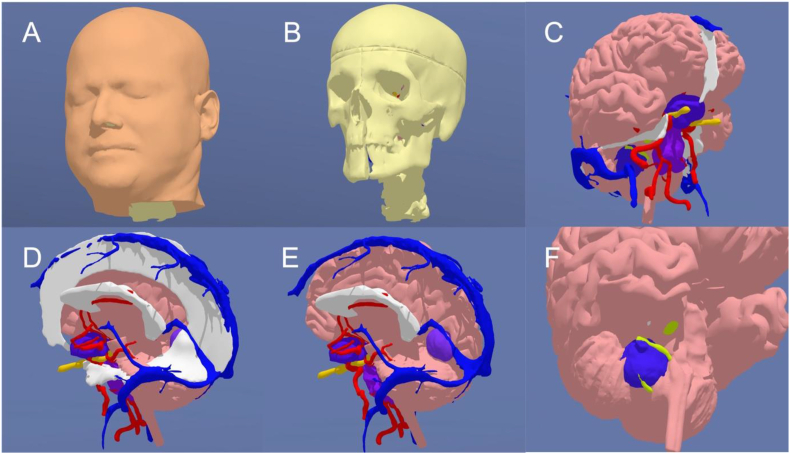
3D-model used for VR simulations and 3D printing, displaying four tumors and the correspondingly modified surrounding anatomy. (A) Skin. (B) Skull and facial bones. (C) Brain parenchyma, dura (including falx cerebri, tentorium, lateral wall of the cavernous sinus), arteries, veins and sinuses, optic nerves (yellow), tuberculum sellae meningioma (dark blue), petroclival chordoma (purple), and vestibular schwannoma (dark blue) surrounding the right facial nerve (neon green). (D) Visualization of the falcotentorial meningioma after removal of the left hemisphere. (E) Visualization of the falcotentorial meningioma after removal of the left hemisphere, falx, and tentorium. (F) Right facial nerve (neon green) surrounded by vestibular schwannoma (dark blue). Once 3D-printed, an electrical wire connected to a light and sound system was implanted in the facial nerve to simulate IONM.

Segmentation and 3D model rendering were performed using the technique described by González-López et al(González-López et al., 2024). Image data was acquired through DICOM files from real patients with tumors (Fig. 1), including CT scans, CT angiography (CTA), and MRI scans. These DICOM files were imported into the segmentation software 3D Slicer version 5.3.0-2023-07-15 (The Slicer Community, http://www.slicer.org). (Fedorov et al., 2012) After segmentation, the 3D objects were refined and converted into triangle-based models using Autodesk MeshMixer version 3.5.474 (Autodesk Inc., San Rafael, CA, USA). The resulting standard triangulated language (STL) files were further processed in Blender® (Blender Foundation, Amsterdam, The Netherlands, www.blender.org) for enhancements, including texture, color, and overall appearance adjustments. Once finalized, the models were utilized for 3D printing (Fig. 3) and virtual reality display (Fig. 4).

3. Hand-Crafted 3D-Printed Head Model

Once the models were prepared, they were physically produced using a 3D printer, following the technique described by González-López et al. (González-López et al., 2024), which is the protocol of the Department of Neurosurgery of the Hospital General Universitario Alicante, Spain, with the collaboration and help of 3DNeurotrainer (www.3dneurotrainer.com). The different parts of the model were printed individually using various materials (depending on the tissue to be created), such as polylactic acid (PLA), acrylonitrile butadiene styrene (ABS), and polyethylene terephthalate glycol (PETG), as well as resins obtained with technologies like stereolithography (SLA) and digital light processing (DLP). Softer materials, such as ballistic gels or silicone, were selected to replicate soft tissues.

The assembly of the different components of a prototype of our model is illustrated in Fig. 3. Four tumors were incorporated into a single model, and the surrounding anatomy was accordingly modified to simulate realistic displacement of neurovascular structures. All cranial nerves, infundibulum and pituitary gland were printed separately. Particular emphasis was placed on accurately replicating the dura mater, allowing for practical dissections that closely mimic the key steps that would be performed in cadaveric specimens (Table 1). The basal dura was created using a thick glue that was sprayed and later hardened to simulate a realistic basal dura that would allow dural reflection to expose the dorsal surface of the anterior clinoid process and thereby perform an extradural anterior clinoidectomy. In addition to the basal dura, the lateral wall of the cavernous sinus was cut out of a rubber sheet and made continuous with the tentorium (of two layers) in the shape and size of the actual structures. The double layers of the tentorium were created to enable an intertentorial dissection after the creation of the cavernous sinus, Meckel's cave and tentorium, and therefore enabled a replication of a transcavernous approach. After printing all tissues namely, bone, nerves, vessels, dura, tumor, and brain, the different parts of the model were assembled by hand to best reflect the actual configuration. The model was also enhanced to allow real time cranial nerve stimulation using electrical currents by the creation of a system with integrated light and sound implanted within the facial nerve to simulate IONM during vestibular schwannoma surgery (Deletis and Fernández-Conejero, 2016; Oh et al., 2012; Rampp et al., 2019; Vivas et al., 2018).

The fabrication of each 3D-printed model for the first course required approximately 4–5 days, including 3D-rendering of each component separately, 3D-printing, molding, and assembly. A total of 13 models were produced for our course. The costs were relatively elevated due to the initial development of segmentation protocols and production workflows. For our next courses, costs will be significantly reduced as the segmentation is now complete, reusable molds and casts are available, and larger-scale production will allow for economies of scale. These improvements are estimated to lower the cost per model, making it more affordable for future courses.

4. Virtual Reality

To supplement the hands-on training, the course also integrated VR environments displayed on HMDs (Fig. 2, Fig. 4). The models were uploaded to the oculus Meta Quest Pro, a VR headset developed by Meta Platforms, Inc., which features built-in sensors and tracking systems (Akehasu et al., 2022). We utilized Gravity Sketch® (London, UK, www.gravitysketch.com), a versatile software utilized for visualizing, modeling, and designing 3D models within a VR environment(Trandzhiev et al., 2023), enabling trainees to explore detailed 3D reconstructions of skull base anatomy and tumor pathologies. This component of the course was designed to enhance spatial understanding and provide an immersive visualization of the surgical steps, allowing participants to review the procedures in a virtual environment before performing them on the 3D-printed model.

5. Cadaveric Dissections

Following the lectures, and prior to the hands-on simulation on the 3D-printed model, a cadaveric dissection session was conducted by an expert faculty who demonstrated the surgical approach under study, guiding the trainees through each surgical step they would later perform on the 3D-printed model (Table 1). This live demonstration allowed for an interactive description of the anatomical landmarks and the surgical techniques.

6. 3D-Printed Model Dissections

The 12 trainees were divided into six pairs, with each pair working together on a model. Each table was supervised by a faculty member, who guided the trainees through the dissection process, offering real-time feedback and expert advice. The instruments used for the dissections were identical to those used in the operating room, including microscope, high-speed drills, cavitronic ultrasonic surgical aspirator (CUSA), microsurgery instruments, and motor stimulation. The only exception was the use of wider suction cannulas, due to the more compact consistency of the debris generated by the 3D-printed materials.

### 2.4 Outcome measures

7. Questionnaires

To evaluate the effectiveness of the course and its educational tools, particularly the hand-crafted 3D-printed model, both trainees and faculty completed questionnaires featuring multiple-choice questions (MCQs) and open-ended feedback sections. Each item was rated on a 5-point scale, where 1 corresponded to “strongly disagree”, 2 to “disagree”, 3 to “neutral”, 4 to “agree”, and 5 to “strongly agree”. The faculty were required to fill out a post-course survey to assess the accuracy, realism, learning objectives, and overall educational value of the models (**see** Appendix A, Appendix A).

8. Self-Assessments

Trainees completed a self-assessment questionnaire both before and after the simulation, assessing their familiarity with anatomical structures, understanding of spatial relationships, knowledge of surgical steps, confidence in performing the procedures, and comfort in handling surgical instruments, rated on a 5-point scale. Additionally, they completed a post-course questionnaire to assess the anatomical accuracy, realism, tactile feedback, educational value, and usability of the hand-crafted 3D-printed model and the VR simulation, and the quality of the lectures and the guidance provided by the faculty (**see** Appendix 1).

### 2.5 Data analysis

The quantitative data from the questionnaires were analyzed using descriptive statistics to provide an overview of participants' evaluations of the course and the educational tools employed. For each item, we calculated the mean (average) and the standard deviation (SD).

The graphical representations of the data in this study were generated using ChatGPT, an artificial intelligence (AI)-based tool developed by OpenAI, in conjunction with Python's data visualization libraries. The AI tool was employed to assist in the creation of figures by organizing and visually presenting the data accurately. All data points, means, and standard deviations were derived from the original datasets collected during the study, and the resulting graphs were carefully reviewed and validated to ensure they meet the publication standards required for this journal. The use of AI in this context was purely for the purpose of streamlining the visualization process, and no AI-generated content was used to interpret or analyze the data.

## 3 Results

A total of 11 faculty members and 12 trainees participated in the course. The faculty comprised 9 males (81.8%) and 2 females (18.2%), with an average age of 47.64 ± 10.54 years and a mean of 25.18 ± 10.46 years of neurosurgical practice. The trainee group consisted of 8 males (66.7%) and 4 females (33.3%), with a mean age of 32.08 ± 4.08 years and an average of 4.70 ± 1.16 years of neurosurgical practice. Participants demographics are summarized in Table 2.

**Table 2:** Participants demographics.

	Faculty Mean (SD)^a^	Trainees Mean (SD)^a^
Total members (number)	11	12
Age (years)	47.64 (10.54)	32.80 (4.08)
Gender		
- Male (n (%))	9/11 (81.8%)	8/12 (66.7%)
- Female (n (%))	2/11 (18.2%)	4/12 (33.3%)
Country (number)		
- Switzerland	6	5
- France	1	2
- Spain	1	1
- Germany	1	0
- Italy	1	0
- Turkey	1	0
- Panama	0	1
- Singapore	0	1
- Czech Republic	0	1
- Belgium	0	1
Neurosurgical practice (years)	25.18 (10.46)	4.70 (1.16)
Current position (number)		
- Senior resident	0	12
- Fellow	3	0
- Attending	6	0
- Head of department	2	0
Previous skull base-dedicated training (number)	1.50 (0.84)	0.20 (0.42)
Number of skull base surgeries (number)		
- Observed	220.00 (168.08)	16.67 (10.80)
- Assisted	370.00 (249.00)	24.00 (13.86)
- First operator	426.00 (609.00)	2.83 (4.02)

a The values are presented as mean and standard deviation (SD), unless stated otherwise.

**Fig. 5:**
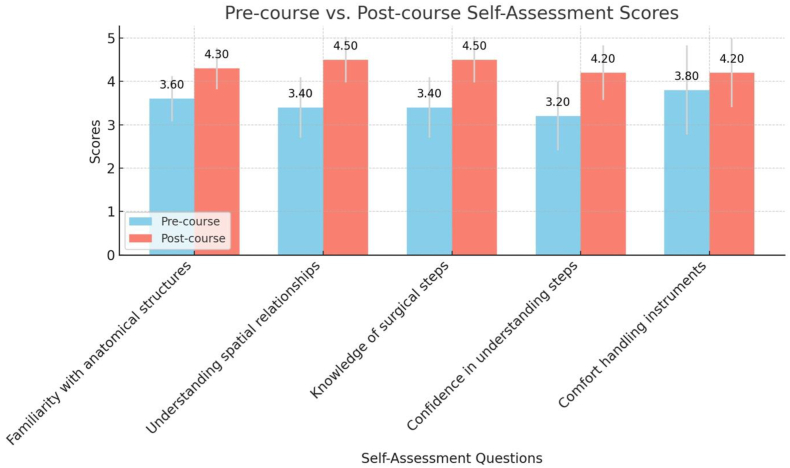
Trainees pre- and post-course self-assessment scores.

The trainees' pre- and post-course self-assessments showed improvement in all areas assessed. The most significant gains were observed in the items “understanding of spatial relationships” and “knowledge of surgical steps”, both of which increased from an average pre-course score of 3.40 ± 0.70 to a post-course score of 4.50 ± 0.53. Furthermore, “confidence in understanding surgical steps” rose from 3.20 ± 0.79 to 4.20 ± 0.63, while “familiarity with anatomical structures” improved from 3.60 ± 0.52 to 4.30 ± 0.48. The smallest improvement was seen in “comfort handling instruments,” which increased from 3.80 ± 1.03 to 4.20 ± 0.79. A summary of the pre- and post-course self-assessment scores is presented in Table 3, with a graphical representation provided in Fig. 5.

**Table 3:** Trainees pre- and post-course self-assessment.

	Pre-course mean score (SD)	Post-course mean score (SD)
I am familiar with the relevant anatomical structures involved in the skull base approaches depicted in this model.	3.60 (0.52)	4.30 (0.48)
I understand the spatial relationships between the key skull base anatomical structures.	3.40 (0.70)	4.50 (0.53)
I know the key surgical steps involved in the performance of the skull base approaches depicted in this model.	3.40 (0.70)	4.50 (0.53)
I am confident in my understanding of the steps involved in the skull base approaches depicted in this model.	3.20 (0.79)	4.20 (0.63)
I am comfortable with handling the instruments used in skull base surgery.	3.80 (1.03)	4.20 (0.79)

The trainees' post-course questionnaire results, summarized in Table 4, assessed overall satisfaction with the hand-crafted 3D-printed head model and educational tools used during the course across seven key areas.

**Table 4:** Trainees post-course questionnaire.

	Mean score (SD)
**Anatomical accuracy**	
The model effectively captured the key anatomical structures involved in the studied skull base approaches.	4.30 (0.82)
The details of the anatomical structures were clear and precise.	4.20 (0.79)
The model effectively captured the displacement of the anatomical structures by the tumor.	4.00 (0.94)
**Realism and tactile feedback**	
The texture and handling of the tissue were realistic compared to actual human tissues.	
- Bone	4.50 (0.53)
- Dura	3.50 (0.97)
- Brain parenchyma	3.60 (0.97)
- Tumor	4.00 (0.67)
- Nerves	3.90 (0.74)
The model accurately replicates the interaction between surgical instruments and tissues.	
- Bone	4.10 (0.88)
- Dura	4.00 (0.47)
- Brain parenchyma	3.60 (1.07)
- Tumor	4.00 (0.94)
- Nerves	3.90 (0.88)
The consistency of the tissue in the model behaved in a realistic way.	
- Bone	4.10 (0.74)
- Dura	3.70 (0.82)
- Brain parenchyma	3.70 (0.95)
- Tumor	3.80 (0.79)
- Nerves	3.90 (0.74)
The positioning of the model and thereby the anatomical structures reflected the actual set-up during surgery.	
- Bone	4.30 (0.67)
- Dura	4.10 (0.74)
- Brain parenchyma	3.80 (0.92)
- Tumor	4.20 (0.79)
- Nerves	4.10 (0.74)
The color code chosen for the tissue was realistic.	
- Bone	4.40 (0.84)
- Dura	4.20 (0.63)
- Brain parenchyma	4.10 (0.74)
- Tumor	3.70 (0.95)
- Nerves	4.00 (0.94)
The neurophysiology simulation was realistic.	4.20 (0.92)
**Educational value**	
The learning objectives were well-aligned with the design and complexity of the model.	4.50 (0.71)
This model serves as a valuable tool for novice neurosurgery residents with limited experience in skull base surgery.	4.60 (0.52)
I would recommend the use of this model to other neurosurgery residents.	4.60 (0.52)
The model effectively helps in developing the necessary skills for skull base surgery.	4.40 (0.70)
The simulation improved my skills.	4.50 (0.71)
The simulation improved my knowledge of surgical neuroanatomy.	4.50 (0.53)
**Usability**	
Overall, the model is easy to set up and use.	4.70 (0.48)
I found it easy to navigate and maneuver within the model.	4.70 (0.48)
**Virtual reality simulation**	
The virtual reality simulation helped me understand the studied skull base approaches.	4.30 (0.67)
**Lectures**	
The microsurgical neuroanatomy of the skull base approaches was well presented.	4.60 (0.84)
The description of the surgical approaches was useful.	4.70 (0.67)
The case description with operative videos and 3D-printed model videos was useful.	4.80 (0.42)
**Faculty**	
The case description with operative videos and 3D-printed model videos was useful.	4.80 (0.42)
The presence of the faculty during the dissections was necessary.	4.80 (0.42)

### 3.1 Anatomical accuracy

Participants evaluated the anatomical accuracy of the model with a mean score of 4.30 ± 0.82 for effectively capturing the key anatomical structures involved in the six studied skull base approaches. The precision of the anatomical details received a mean score of 4.20 ± 0.79, while the realism of the displacement of structures by tumors was rated 4.00 ± 0.94.

### 3.2 Realism and tactile feedback

Participants rated the realism and tactile feedback of the model across several subcategories.

The texture of the bone was given a mean score of 4.50 ± 0.53, while the dura texture was rated 3.50 ± 0.97. The brain parenchyma texture received a score of 3.60 ± 0.97, the tumor texture was rated 4.00 ± 0.67, and the nerves texture scored 3.90 ± 0.74.

For the interaction between surgical instruments and tissues, the bone received a rating of 4.10 ± 0.88, dura 4.00 ± 0.47, brain parenchyma 3.60 ± 1.07, tumor 4.00 ± 0.94, and nerves 3.90 ± 0.88.

Regarding tissue consistency, bone was rated 4.10 ± 0.74, dura 3.70 ± 0.82, brain parenchyma 3.70 ± 0.95, tumor 3.80 ± 0.79, and nerves 3.90 ± 0.74.

The positioning of the anatomical structures in the model, in terms of reflecting the actual surgical setup, was rated 4.30 ± 0.67 for bone, 4.10 ± 0.74 for dura, 3.80 ± 0.92 for brain parenchyma, 4.20 ± 0.79 for tumor, and 4.10 ± 0.74 for nerves.

The color coding used for the tissues received the following ratings: bone 4.40 ± 0.84, dura 4.20 ± 0.63, brain parenchyma 4.10 ± 0.74, tumor 3.70 ± 0.95, and nerves 4.00 ± 0.94.

Finally, the neurophysiology simulation was rated 4.20 ± 0.92.

### 3.3 Educational value

In the educational value category, the alignment of learning objectives with the design and complexity of the model received a mean score of 4.50 ± 0.71. The model was rated 4.60 ± 0.52 in terms of its value for novice neurosurgery residents with limited experience in skull base surgery. Additionally, participants rated their likelihood of recommending the model to other neurosurgery residents at 4.60 ± 0.52. The effectiveness of the model in helping develop the necessary skills for skull base surgery was rated 4.40 ± 0.70, and the impact of the simulation on improving skills and knowledge of surgical neuroanatomy was rated 4.50 ± 0.71 and 4.50 ± 0.53, respectively.

### 3.4 Usability

For usability, participants rated the ease of setting up and using the model at 4.70 ± 0.48. The ease of navigating and maneuvering within the model also received a score of 4.70 ± 0.48.

### 3.5 Virtual reality simulation

The VR simulation, designed to aid in understanding the studied skull base approaches, was rated 4.30 ± 0.67 by the participants.

### 3.6 Lectures

Participants rated the lectures and faculty involvement during the course. The presentation of the microsurgical neuroanatomy of the skull base approaches received a mean score of 4.60 ± 0.84. The description of the surgical approaches was rated 4.70 ± 0.67. The case descriptions, which included operative videos and 3D-printed model videos, were rated 4.80 ± 0.42.

### 3.7 Faculty

The usefulness of the presence of faculty and their guidance during dissections was rated 4.80 ± 0.42.

**Fig. 6:**
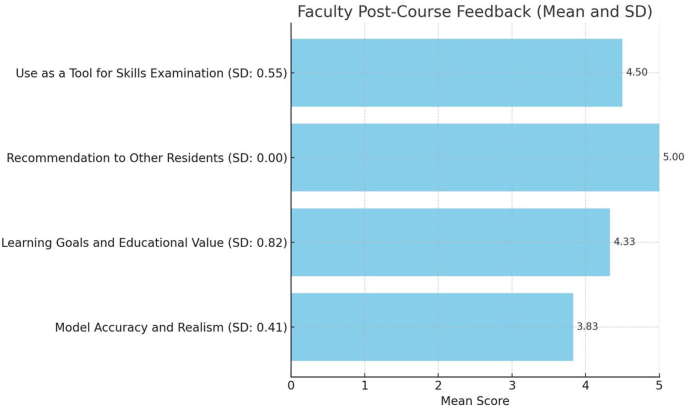
Faculty post-course questionnaire.

Finally, the results of the faculty post-course questionnaire focused on the model's accuracy, realism, and educational value, are detailed in Table 5, and visualized in Fig. 6. The model's ability to effectively capture the key anatomical features of the skull base approaches was rated 3.83 ± 0.41, and the texture and responsiveness of the tissues received a rating of 3.83 ± 0.98.

**Table 5:** Faculty post-course questionnaire.

	Mean score (SD)
**Model accuracy and realism**	
The model effectively captured the key anatomical features involved in the studied skull base approaches.	3.83 (0.41)
The texture and responsiveness of the tissues in the model closely mimicked those of real tissue.	3.83 (0.98)
**Learning goals and educational value**	
The learning objectives were well-aligned with the design and complexity of the model.	4.33 (0.82)
This model serves as a valuable tool for novice neurosurgery residents with limited experience in skull base surgery.	4.83 (0.41)
I would recommend the use of this model to other neurosurgery residents.	5.00 (0.00)
This model could serve as a tool for neurosurgical skills examination.	4.50 (0.55)

For learning goals and educational value, the alignment of the learning objectives with the model's design and complexity was rated 4.33 ± 0.82 by the faculty. The model as a tool for novice neurosurgery residents was rated 4.83 ± 0.41. The recommendation of the model to other neurosurgery residents received a score of 5.00 ± 0.00. The model's potential as a tool for neurosurgical skills examination was rated 4.50 ± 0.55.

## 4 Discussion

Our study found that both trainees and faculty rated the 3D-printed head model highly for its educational value and usability. The faculty specifically noted the potential for these models to serve as valuable tools in the examination of surgical skills (e.g., in board exams or internal residents’ evaluation), with an overall rating of 4.50 ± 0.55, and recommended them to other residents with a perfect score of 5.00 ± 0.00. Trainees reported significant improvements across all tested items, with the highest improvement in understanding spatial relationships, reflected by self-assessment scores increasing from 3.40 ± 0.70 to 4.50 ± 0.53 after the course. The use of VR was also rated highly, with an average score of 4.30 ± 0.67.

In addition to the primary findings, secondary results highlighted that the tactile feedback for soft tissues, such as dura and brain parenchyma, were areas of improvement. With average scores of 3.50 ± 0.97 and 3.60 ± 0.97, respectively, these results suggest that the material realism and the overall simulation of these tissues could be enhanced for a more accurate representation.

These findings align with existing literature that highlights the role of 3D-printed models in enhancing surgical planning and resident education by offering a more immersive and hands-on learning experience compared to 2D imaging or traditional textbooks (Muelleman et al., 2015; D'Urso et al., 1999). Studies show that 3D-printed models can improve spatial understanding and procedural planning, especially in complex areas like skull base surgery, where anatomical relationships are difficult to visualize in three dimensions (Kondo et al., 2016). In our course, trainees reported significant improvements in understanding spatial relationships, further supporting the notion that 3D-printed models help bridge the gap between theoretical knowledge and hands-on practice (Su et al., 2018). Moreover, one of the key strengths of these models is their ability to replicate not only patient-specific anatomy, but also pathologies such as brain tumors or cerebrovascular diseases.

Access to cadaveric heads requires expensive techniques, specialized equipment, and materials to ensure proper preservation, which is one of the key limitations of this educational method (Rajasekhar and Dinesh, 2021; Okafor et al., 2023). This has contributed to a shift toward using alternatives like 3D models and virtual simulations in medical schools. Although the initial production process of 3D-printed models can be time-consuming and costly, especially when replicating soft tissues like brain parenchyma or dura with high fidelity (Tai et al., 2016), the costs decrease significantly once the first model is produced. As we experienced while building our handcrafted head model, several adjustments and trials with different materials and techniques were necessary. However, once the first model is perfected, casts can be made for subsequent models, reducing overall costs and addressing the financial challenges associated with acquiring cadaveric heads. Although using these pathological models for preparation for specific and complex surgical procedures may be realistic, the time-consuming and costly process of designing the model, selecting materials, and assembly would be difficult to justify if the model is only used for one patient's procedure.

Institutional challenges in obtaining cadaveric specimens for educational purposes are significant. One major issue is the scarcity of donations, exacerbated by legal, ethical, and logistical barriers. For example, the COVID-19 pandemic exacerbated shortages in medical schools, affecting anatomy education and surgical training programs globally (Rajasekhar and Dinesh, 2021). Strict regulations governing body donation, such as those outlined in most legal frameworks, add complexity to the procurement process (Rajasekhar and Dinesh, 2021; George and De, 2010). These limitations have also led to the use of complementary educational methods, such as 3D models (Okafor et al., 2023).

Despite their numerous advantages, 3D-printed models still face some challenges. The quality of the models is heavily dependent on the accuracy of medical imaging data, the software used for segmentation and rendering, and the realism of material used for printing. These issues were also highlighted in other studies, where the complexity of rendering soft tissue in a realistic way was identified as a key limitation (Narayanan et al., 2015). In our course, the tactile feedback for tissues like dura and brain parenchyma received lower scores, which aligns with previous research indicating that soft-tissue simulation remains an area for improvement (Lee et al., 2022).

Recent research has demonstrated the growing potential of VR in neurosurgical training. For example, a study by Lai et al. (2023) validated the use of VR for the middle cranial skull base approach. Their findings confirm that VR can significantly enhance both anatomical comprehension and surgical precision, making it a valuable complement to traditional methods such as cadaveric dissection and live surgery. This highlights the effectiveness of VR as an educational tool, offering a safe, cost-effective, scalable, and controlled environment for skill acquisition. Given the results of our study, which showed similarly high ratings for 10.13039/501100004359VR (4.30 ± 0.67), it is evident that VR continues to prove its utility in neurosurgical training, as also supported by recent literature (Neyem et al., 2024; Sandralegar et al., 2024; Mirchi et al., 2020).

## 5 Limitations

The small sample size of 12 trainees and 11 faculty members restricts the generalizability of the results. Additionally, the subjective nature of self-assessments may introduce bias, making it challenging to fully assess the objective effectiveness of the training tools. Finally, the study did not assess the long-term impact of these tools on surgical skill development or patient outcomes, which would be valuable for future research.

## 6 Recommendations

Based on our findings, we recommend incorporating pathological 3D-printed head models and VR into the training curriculum for skull base surgery as complementary tools to traditional cadaveric dissections. These models have demonstrated significant potential in enhancing the understanding of complex anatomical structures and surgical procedures, as noted by both trainees and faculty in our study. Additionally, we propose that 3D-printed models could be integrated into board examinations and internal residents' evaluations, as emphasized by faculty members in our study. Furthermore, these courses could be incorporated into national or European skull base surgery training modules, allowing for standardization in neurosurgical education and ensuring more residents have access to these advanced learning tools.

## 7 Conclusion

The integration of VR and head models using 3D printing in neurosurgical training offers a promising approach to improving the educational experience for trainees. While some limitations remain, particularly regarding the tactile feedback of soft tissues, advancements in technology are likely to mitigate these issues in the near future. The use of these tools not only enhances anatomical understanding and surgical technique but also provides a cost-effective, accessible, and realistic environment for skill development, which is essential for complex procedures like skull base surgery.

## Declaration of competing interest

The authors declare that they have no known competing financial interests or personal relationships that could have appeared to influence the work reported in this paper.

## Footnotes

Appendix A Supplementary data to this article can be found online at https://doi.org/10.1016/j.bas.2024.104163.
