# Supplementary material for: Evaluating the impact of a hand-crafted 3D-Printed head Model and virtual reality in skull base surgery training
Source: Brain Spine. 2024 Dec 12;5:104163. doi: 10.1016/j.bas.2024.104163 (PMC11718289; doi:10.1016/j.bas.2024.104163)
Supplement: Multimedia component 1 [file mmc1.pdf]

COURSE EVALUATION - TRAINEES

1. General information

**Name**		
**Sex (male/female)**		
**Age (years)**		
**Hospital**		
**Years of neurosurgical training**		
**Current position (junior resident/senior resident/fellow/attending)**		
**Previous skull base-dedicated training (e.g., fellowship)**		
**Dominant hand (left/right/ambidextrous)**		
**Number of previous experiences with 3D-printed models in neurosurgical training**		
**Number of skull base surgeries**	Observed	
	Assisted	
	First operator	

2. Pre- and post-course questionnaires completed by trainees

1 = Strongly disagree
2 = Disagree
3 = Neutral
4 = Agree
5 = Strongly agree

	1	2	3	4	5
**PRE-COURSE**					
**Pre-course assessment**					
I am familiar with the relevant anatomical structures involved in the skull base approaches depicted in this model.					
I understand the spatial relationships between the key skull base anatomical structures.					
I know the key surgical steps involved in the performance of the skull base approaches depicted in this model.					
I am confident in my understanding of the steps involved in the skull base approaches depicted in this model.					
I am comfortable with handling the instruments used in skull base surgery.					

**POST-COURSE**					
**Anatomical accuracy**					
The model effectively captured the key anatomical structures involved in the studied skull base approaches.					
The details of the anatomical structures were clear and precise.					
The model effectively captured the displacement of the anatomical structures by the tumor.					
**Realism and tactile feedback**					
- **Bone**					
The texture and handling of the tissues were realistic compared to actual human tissues.					
The model accurately replicates the interaction between surgical instruments and tissues.					
The consistency of the bone in the model behaved in a realistic way.					
The positioning of the model and thereby the anatomical structures reflected the actual set-up during surgery.					
The color code chosen for the anatomical structures was realistic.					
- **Dura**					
The texture and handling of the tissues were realistic compared to actual human tissues.					
The model accurately replicates the interaction between surgical instruments and tissues.					
The consistency of the dura in the model behaved in a realistic way.					

The positioning of the model and thereby the anatomical structures reflected the actual set-up during surgery.					
The color code chosen for the anatomical structures was realistic.					
- **Brain parenchyma**					
The texture and handling of the tissues were realistic compared to actual human tissues.					
The model accurately replicates the interaction between surgical instruments and tissues.					
The consistency of the brain in the model behaved in a realistic way.					
The positioning of the model and thereby the anatomical structures reflected the actual set-up during surgery.					
The color code chosen for the anatomical structures was realistic.					
- **Nerves**					
The texture and handling of the tissues were realistic compared to actual human tissues.					
The model accurately replicates the interaction between surgical instruments and tissues.					
The consistency of the nerves in the model behaved in a realistic way.					
The positioning of the model and thereby the anatomical structures reflected the actual set-up during surgery.					
The color code chosen for the anatomical structures was realistic.					
The neurophysiology simulation was realistic.					
- **Tumor**					
The texture and handling of the tissues were realistic compared to actual human tissues.					
The model accurately replicates the interaction between surgical instruments and tissues.					
The consistency of the tumors in the model behaved in a realistic way.					
The positioning of the model and thereby the anatomical structures reflected the actual set-up during surgery.					
The color code chosen for the anatomical structures was realistic.					
**Educational value**					
The learning objectives were well-aligned with the design and complexity of the model.					
This model serves as a valuable tool for novice neurosurgery residents with limited experience in skull base surgery.					
I would recommend the use of this model to other neurosurgery residents.					
The model effectively helps in developing the necessary skills for skull base surgery.					
The simulation improved my skills.					
The simulation improved my knowledge of surgical neuroanatomy.					
**Usability**					
Overall, the model is easy to set up and use.					
I found it easy to navigate and maneuver within the model.					
**Virtual reality simulation**					

The virtual reality simulation helped me understand the studied skull base approaches.					
**Post-course assessment**					
I am familiar with the relevant anatomical structures involved in the skull base approaches depicted in this model.					
I understand the spatial relationships between the key skull base anatomical structures.					
I know the key surgical steps involved in the performance of the skull base approaches depicted in this model.					
I am confident in my understanding of the steps involved in the skull base approaches depicted in this model.					
I am comfortable with handling the instruments used in skull base surgery.					
**Lectures**					
The microsurgical neuroanatomy of the skull base approaches was well presented.					
The description of the surgical approaches was useful.					
The case description with operative videos and 3D-printed model videos was useful.					
**Faculty**					
The faculty guided me to perform the studied skull base approaches on the model.					
The presence of the faculty during the dissections was necessary.					

3. Open-ended feedback

Please provide any additional comments or suggestions regarding the course.
