# Supplementary material for: Evaluating the impact of a hand-crafted 3D-Printed head Model and virtual reality in skull base surgery training
Source: Brain Spine. 2024 Dec 12;5:104163. doi: 10.1016/j.bas.2024.104163 (PMC11718289; doi:10.1016/j.bas.2024.104163)
Supplement: Multimedia component 2 [file mmc2.pdf]

THE EUROPEAN ASSOCIATION OF NEUROSURGICAL SOCIETIES

1st LAUSANNE NET Lab SKULL BASE COURSE
3D PRINTED HEAD MODELS WITH TUMORS

COURSE EVALUATION - FACULTY

1. General information

**Name**		
**Sex (male/female)**		
**Age (years)**		
**Hospital**		
**Years of neurosurgical training**		
**Current position (junior resident/senior resident/fellow/attending)**		
**Previous skull base-dedicated training (e.g., fellowship)**		
**Dominant hand (left/right/ambidextrous)**		
**Number of previous experiences with 3D-printed models in neurosurgical training**		
**Number of skull base surgeries**	Observed	
	Assisted	
	First operator	

2. Post-course questionnaire completed by faculty

1 = Strongly disagree
2 = Disagree
3 = Neutral
4 = Agree
5 = Strongly agree

	1	2	3	4	5
**Model accuracy and realism**					
The model effectively captured the key anatomical features involved in the studied skull base approaches.					
The texture and responsiveness of the tissues in the model closely mimicked those of real tissue.					
**Learning goals and educational value**					
The learning objectives were well-aligned with the design and complexity of the model.					
This model serves as a valuable tool for novice neurosurgery residents with limited experience in skull base surgery.					
I would recommend the use of this model to other neurosurgery residents.					
This model could serve as a tool for neurosurgical skills examination.					

3. Open-ended feedback

Please provide any additional comments or suggestions regarding the course.
